# Atypical Presentation of Plasmacytoma in a Young Woman

**DOI:** 10.7759/cureus.72787

**Published:** 2024-10-31

**Authors:** Mark Sahyouni, James J Cappola

**Affiliations:** 1 Internal Medicine, Campbell University School of Osteopathic Medicine, Lillington, USA

**Keywords:** bone marrow biopsy (bmb), lytic lesion of rib, rare condition of the rib, rib, solitary bone plasmacytoma

## Abstract

A 45-year-old woman with a history of hypertension, polycystic ovary syndrome, and a family history of triple-negative breast cancer presented to our primary care clinic with an incidental fifth rib mass on a computed tomography (CT) scan of the chest. She reported mild intermittent left-sided chest pain and no constitutional symptoms. One year prior, she had a workup for a breast lesion, which was negative for breast cancer. Two months later, a repeat chest CT and a skeletal bone survey revealed an expansile and destructive left fifth rib lytic lesion concerning multiple myeloma (MM) but no other bony lesions. Serum protein electrophoresis revealed elevated IgA levels, free kappa light chains, and an M spike. A bone marrow biopsy showed an increase in plasma cells but with no clonality. A whole-body positron emission tomography (PET) scan was negative for other bony lesions or other solid tumors. A CT-guided biopsy of the left fifth rib revealed a solitary plasmacytoma (SP). This case study portrays a rare site for an SP and raises clinical awareness for the condition in patients presenting with atypical chest pain. The patient underwent localized radiation therapy, which is the mainstay of treatment for patients with this condition. Patients should also be monitored periodically for progression to MM if diagnosed and treated for an SP.

## Introduction

A solitary plasmacytoma (SP) can be defined as a single clonal plasma cell tumor with or without bone marrow degeneration and variation of symptoms [1,2]. Complications of SPs include bone pain, fractures, soft tissue injury, neurological damage, or neuropathy [3]. SPs can present in different ways in bony tissues or extramedullary (in soft tissues) [1]. Plasmacytomas are considered an intermediate phase of malignancy between monoclonal gammopathy of undetermined significance (MGUS) and multiple myeloma (MM), making it crucial to correctly identify, diagnose, and treat [4]. There is no definitive cause of SPs, but the literature suggests there are risk factors such as genetics, exposure to radiation, and long-term antigenic stimulation [5].

The annual incidence of SP is 0.15/100,000, or fewer than 450 cases in the United States [1,4]. SPs occur in bony tissue in 70% of patients; the remaining patients have extramedullary SPs [1]. Although SPs can occur in any bone, they are commonly found in the skull and spine and less commonly in the scapula, clavicle, and ribs [1]. They tend to occur more often inside bone marrow than out. Demographically, SPs occur in men more commonly than females (2:1 ratio), with a mean age of 55-60 years old [4].

Patients typically complain of bone pain in the cervical or lumbar spine, femur, pelvis, and/or ribs [4]. SPs in the ribs tend to be the rarest from the sites listed and lack definite criteria for diagnosis and treatment [1]. For other sites, diagnostic criteria for SPs include these four characteristics: (1) single lesion of bone or soft tissue; (2) no clonal plasma cells in the bone marrow; (3) no additional lesions from whole body imaging; and (4) no end-organ damage, hypercalcemia, or anemia [1].

A bone marrow biopsy is warranted in a patient with a suspected SP to exclude MM. A bone marrow biopsy excludes MM if there are less than 10% monoclonal plasma cells on immunophenotyping by kappa/lambda typing and the presence of CD138 and CD38 markers [2,4]. Less invasive diagnostics, including imaging, are commonly used to view the painful area of interest the patient may be having trouble with. Radiographic imaging shows a predominantly lytic pattern more than two-thirds of the time, whereas the other third can be multi-cystic in nature [2].

Recommended imaging for a suspected SP includes computed tomography (CT), magnetic resonance imaging (MRI), or positron emission tomography (PET) scans due to their higher sensitivity than radiographs [2]. Studies have found CT/PET scans were significantly more specific than MRI for detecting SPs [2]. The specificity for CT/PET was 99% versus 89% for MRI [2].

As mentioned earlier, SPs have the propensity to transform into MM. A study by Paiva et al. found that nearly 50% of SPs had BM clonal plasma cells [6]. Among patients with an SP with clonal plasma cells in bone marrow, 71% progressed to MM [6]. Only 8% of patients with an SP without clonal plasma cells in bone marrow progressed to MM [6]. The median time to MM progression was 26 months among patients with an SP with BM clonal plasma cells [6].

Another study by Ohana et al. found that patients with an SP with BM clonal plasma cells had a 20-60% risk of developing MM within three years [7]. Patients with an SP without BM clonal plasma cells had a 10% risk of MM in three years [7].

## Case presentation

The patient is a 45-year-old woman with a history of hypertension, polycystic ovary syndrome, and a family history of triple-negative breast cancer. The patient was first seen at our primary care clinic following an emergency department (ED) visit for pneumonia in which an incidental left fifth rib mass was found on a chest CT. She was referred to cardiothoracic surgery, and a PET scan was ordered. Unfortunately, the patient’s insurer denied coverage for the PET scan, and the patient was lost to follow up for about 19 months. The patient did have a suspicious breast lesion on the screening mammogram, but a subsequent biopsy ruled out breast cancer within that period and lost follow-up.

**Figure 1:**
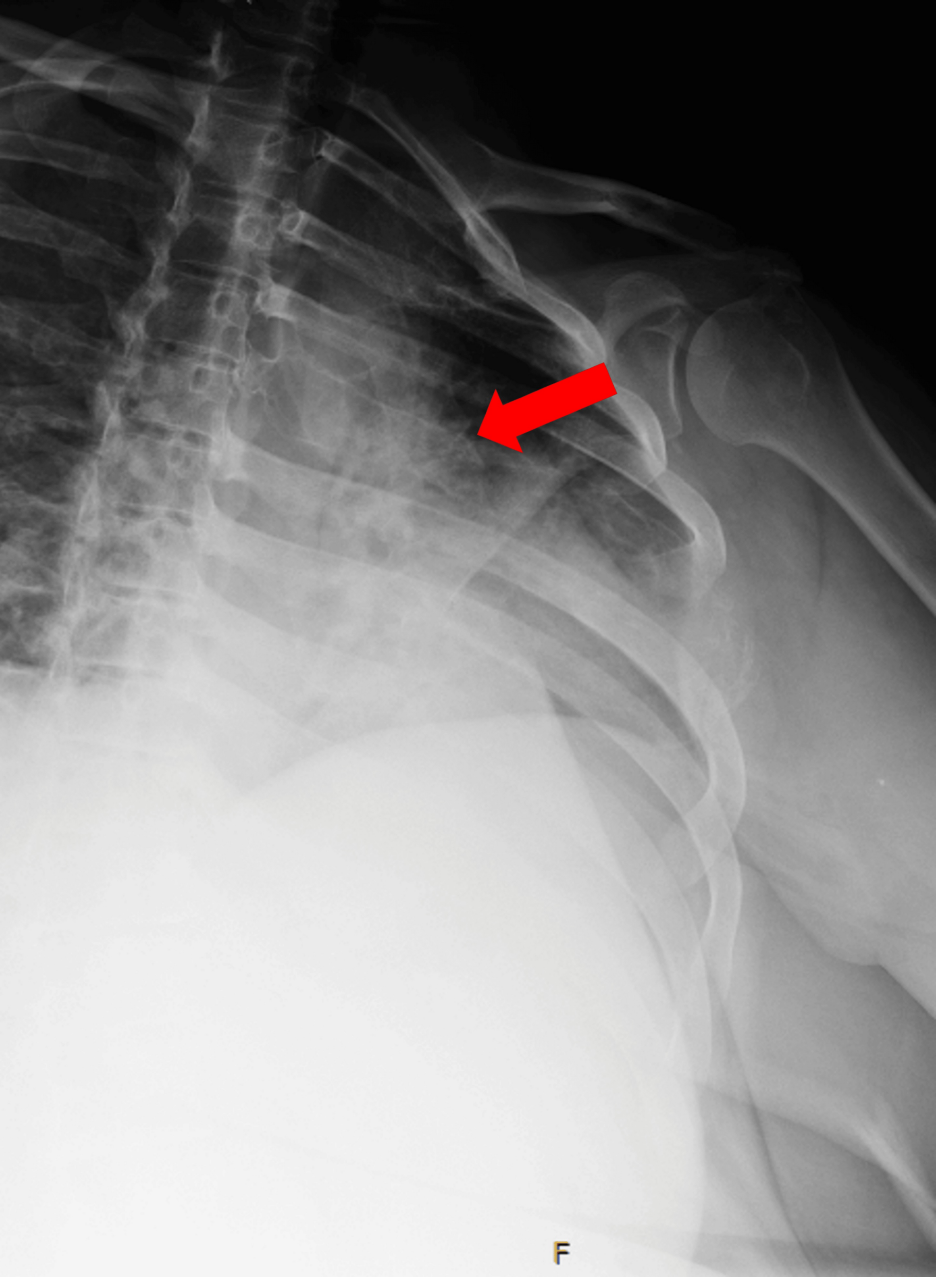
Destructive lytic mass on left fifth rib

**Figure 2:**
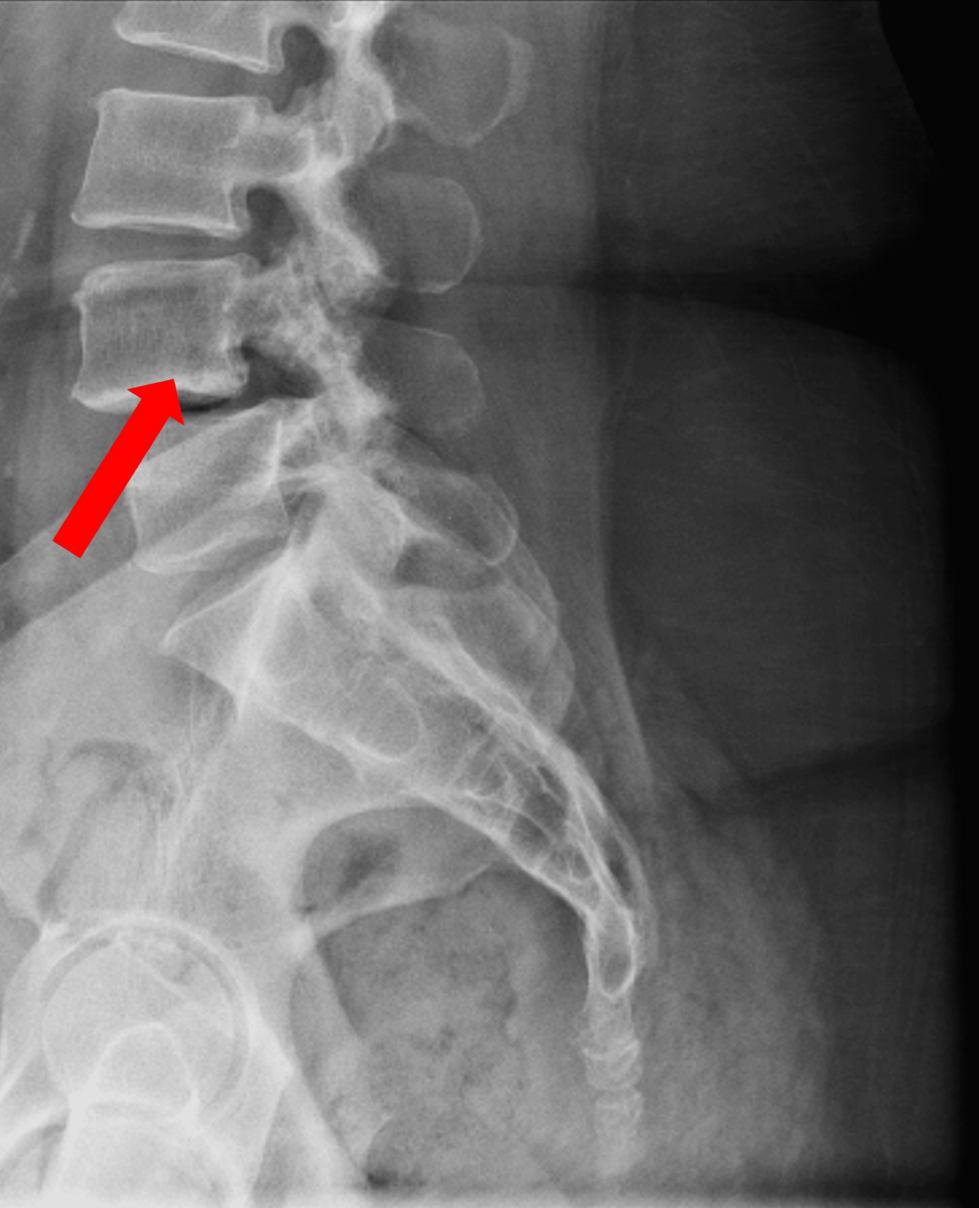
Possible lytic process in L4 vertebral body

About 19 months later, she returned to the clinic after another ED encounter for lower back pain. The patient was picking up her child and heard a “pop” in her lower back that was painful. She reported mild intermittent left-sided chest pain, usually exacerbated by sneezing. She had no other neurologic symptoms or neurologic deficits on the physical exam. A chest X-ray revealed an enlarging destructive lytic mass on the left fifth rib (Figure 1). A lumbar X-ray showed a possible lytic process in the L4 vertebral body (Figure 2).

One month later, on her return visit to the clinic, a serum protein electrophoresis (SPEP) revealed elevated alpha 2 globulin levels with normal amounts of protein (Table 1). Additional labs for inflammatory markers were also ordered (Table 2).

**Table 1:** Results from SPEP SPEP: serum protein electrophoresis

SPEP	Patient’s laboratory value	Reference range
Protein	7.6 g/dL	6.0-8.3 g/dL
Alpha 1 globulin	0.30 g/dL	0.19-0.46 g/dL
Alpha 2 globulin	1.10 g/dL (H)	0.48-1.05 g/dL
Beta globulin	0.48 g/dL	0.48-1.10 g/dL
Gamma region	1.40 g/dL	0.62-1.51 g/dL

**Table 2:** Results for inflammatory markers

Laboratory test	Patient’s laboratory value	Reference range
Lactate dehydrogenase	125 U/L (L)	135-275 U/L
C-reactive protein	13 mg/L (H)	<5 mg/L
Sedimentation rate	29 mm/hr (H)	0-20 mm/hr

**Figure 3:**
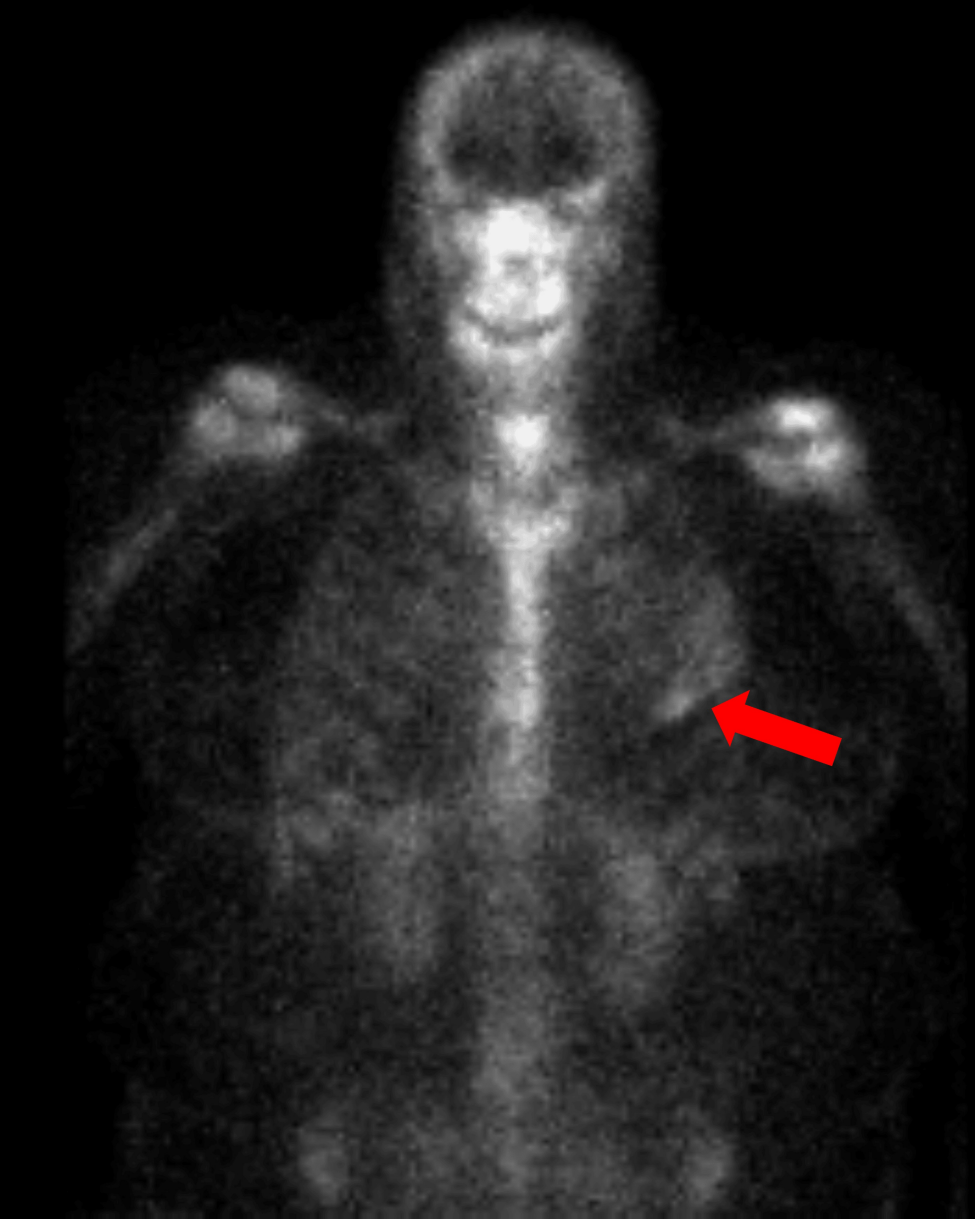
Abnormal tracer termination of left fifth rib via nuclear medicine body whole body scan

**Figure 4:**
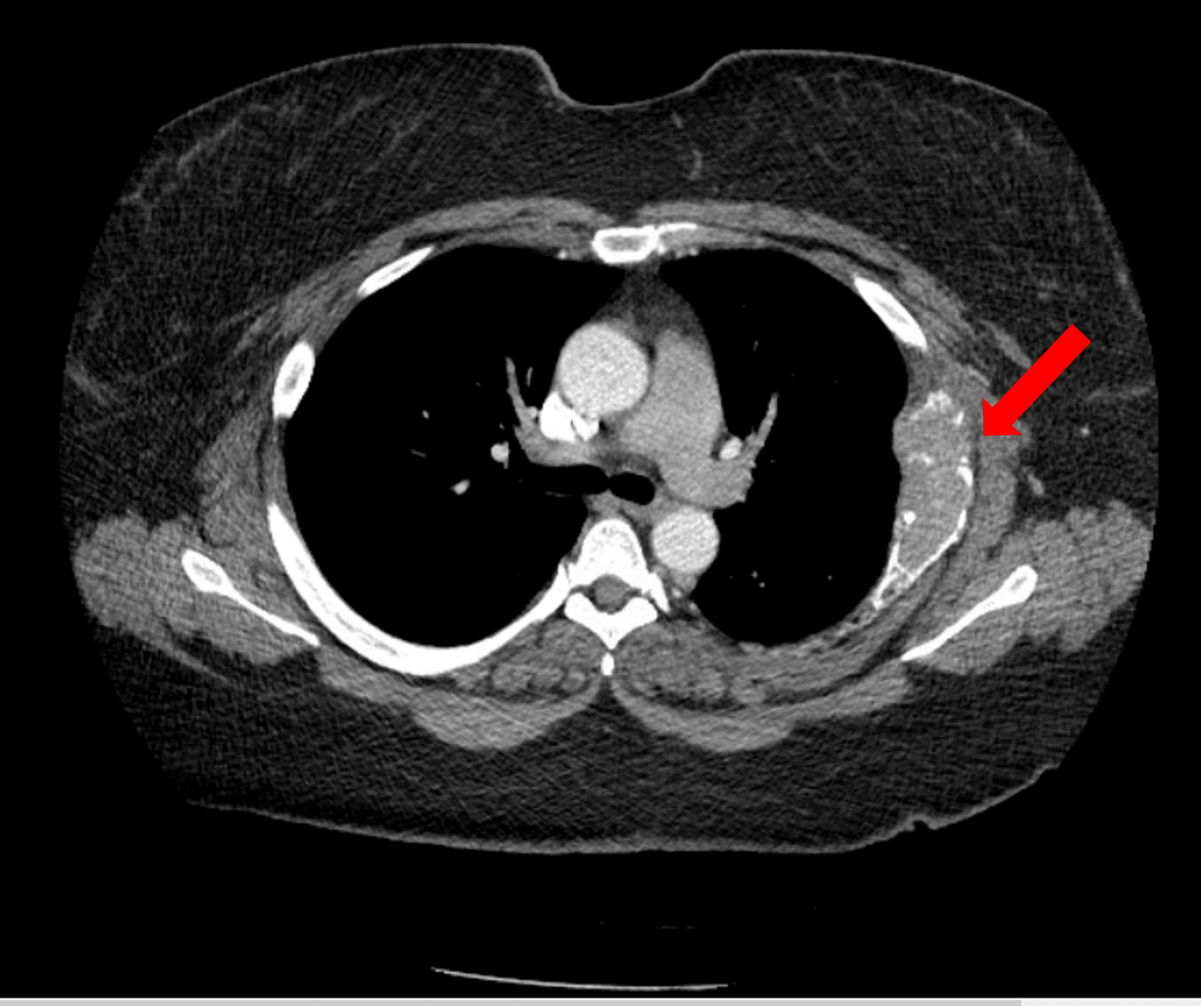
CT scan with contrast displaying an expansive osseous lesion of the left fifth rib

**Figure 5:**
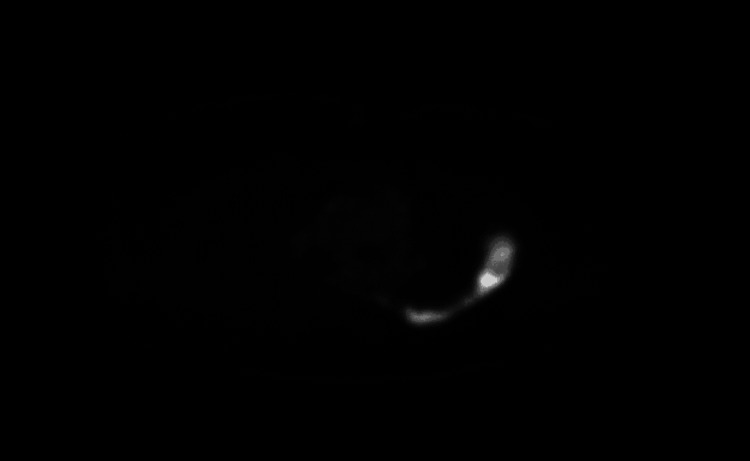
PET scan portraying an expansile left fifth rib lytic lesion

One month later, the patient underwent a nuclear medicine bone whole-body scan (Figure 3) that showed abnormal tracer termination within the left anterior fifth rib correlated with an abnormal bone lesion. The patient was referred to a hematologist/oncologist who obtained an immunofixation electrophoresis, protein electrophoresis, urine protein electrophoresis, and kappa/lambda free light chains (Table 3). A chest CT (Figure 4) revealed an expansive osseous lesion of the left fifth rib. A whole-body PET scan (Figure 5) showed increased activity in the left fifth rib but no other lytic or blastic bone lesions or other solid tumors.

**Table 3:** Results for multiple myeloma panel

Multiple myeloma panel	Patient’s laboratory value	Reference range
IgG	1422 mg/dL	586-1602 mg/dL
IgA	381 mg/dL (H)	87-352 mg/dL
IgM	92 mg/dL	26-217 mg/dL
Monoclonal (M) spike	0.8 g/dL (H)	0 mg/dL
Monoclonal (M) spike (urine)	5.5% (H)	0%
Free kappa light chains	107.4 mg/L (H)	3.3-19.4 mg/L
Free lambda light chains	20.4 mg/L	5.7-26.3 mg/L
Beta-2-microglobulin	1.8 mg/L	0.6-2.4 mg/L

**Figure 6:**
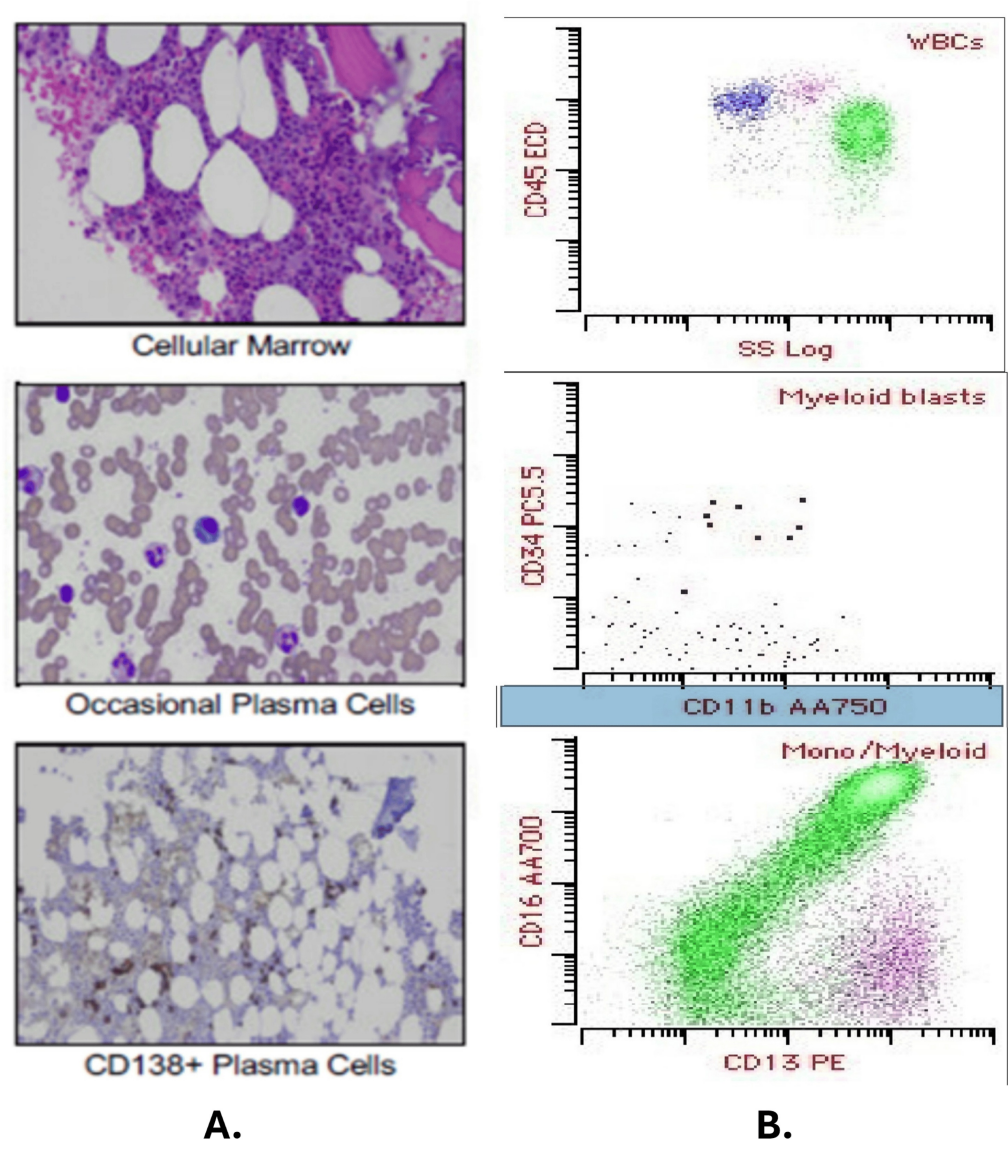
BMB displaying CD138+ plasma cells (A) and flow cytometry resulting in no monoclonal plasma cell population (B) BMB: bone marrow biopsy

A bone marrow biopsy with flow cytometry (Figure 6) was performed. A bone marrow biopsy showed a mild increase in plasma cells that appeared polyclonal. Fluorescence in situ hybridization (FISH) studies were negative for plasma cell neoplasm-associated genetic abnormalities. Additionally, the flow cytometry showed no monoclonal b-cell population or immunophenotypically abnormal t-cell populations.

**Figure 7:**
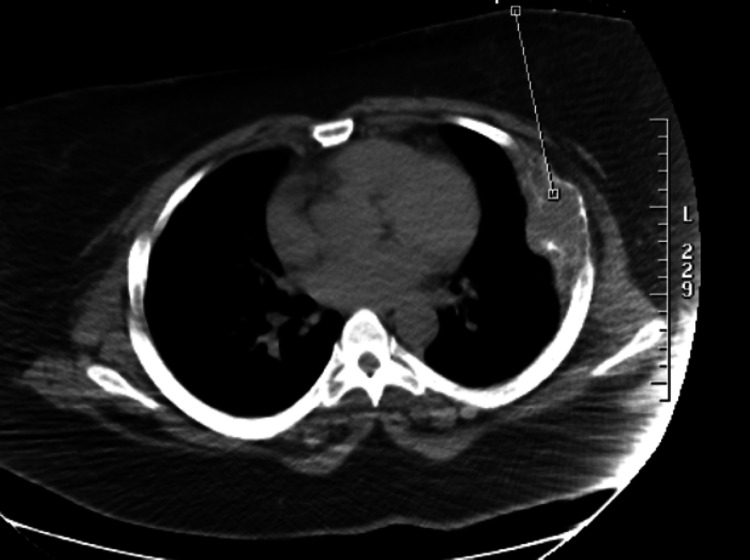
CT-guided left fifth rib biopsy

About two years following her initial presentation, a second rib biopsy was obtained that yielded “marrow cellularity is diffusely reactive for CD138 and kappa light chain without significant staining for lambda light chain" (Figure 7). These findings, in addition to her imaging, which ruled out any other solid tumors, and her bone marrow biopsy, which ruled out an increase in monoclonal plasma cells, confirmed the diagnosis of SP.

A referral was made to radiation oncology for treatment of the lesion. Our patient began radiation therapy with 5000 centigray (cGy) in 25 fractions scheduled to begin the following month.

## Discussion

The median survival time of patients with a SP is 10.7 years, with 5-, 10-, and 20-year survival rates of 75%, 52%, and 37%, respectively [8]. The following prognostic factors for SPs are associated with poor outcomes: (1) 40 years or older, (2) lesion > 5 cm, (3) lesions involving the spine, (4) presence of light chains, (5) elevated M protein levels in serum, (6) persistence of M proteins after treatment, (7) clonal plasma cell bone marrow involvement, (8) associated neurologic symptoms, and/or (9) osteopenia [1,2,4]. Another complication is relative immunodeficiency due to defective immunoglobulin function via decreased immune response to antigens [3]. Last, end-organ damage in the form of amyloidosis can result from light chain deposition in the renal parenchyma [3].

The main treatments for SPs are localized radiation and, rarely, surgical resection [1,2]. Chemotherapy should not be considered as a solo treatment modality for SPs. Iqbal and Majid cite studies that revealed no disease control or decrease in complications when SPs were treated with chemotherapy alone [4]. Although chemotherapy does not prevent the progression of SPs to MM, it may delay it. A study by Tsang found a 100% control rate of SPs with chemotherapy alone with tumors less than 5 cm but only 38% control when with tumors greater than 5 cm [9]. Therefore, Iqbal and Majid suggest that chemotherapy can be used in conjunction with radiation or surgery when the tumor is greater than 5 cm and does not respond to radiation [4].

The only other treatment modality is surgical removal of the lytic lesion. Surgery is indicated for tumors causing spine fractures, in which case decompressive laminectomy and stabilization of the spine are indicated [10]. Possible surgical methods include vertebroplasty and kyphoplasty to fix fractures or resultant neurological complications [10]. Surgery should be utilized as an adjuvant to radiation for best results, as there is a high likelihood of recurrence without it [10].

## Conclusions

This study adds evidence to the paucity of literature on the diagnosis and management of an SP at a rare site. As previously mentioned, SP in the rib is the rarest of possible sites where they tend to appear. In patients with atypical chest/rib pain, it may be prudent to investigate the pain with labs and/or imaging. In primary care settings, it is suggested to start with ordering a complete blood count with differential and an SPEP. This can be followed with imaging modalities such as CT or PET scans to identify SP if anything appears abnormal with the lab results. It is imperative to diagnose and treat plasmacytomas early and to monitor patients for bone marrow involvement and progression to MM.
